# The Presence of Myosteatosis Is Associated with Age, Severity of Liver Disease and Poor Outcome and May Represent a Prodromal Phase of Sarcopenia in Patients with Liver Cirrhosis

**DOI:** 10.3390/jcm12093332

**Published:** 2023-05-07

**Authors:** Eleni Geladari, Theodoros Alexopoulos, Meropi D. Kontogianni, Larisa Vasilieva, Iliana Mani, Roxane Tenta, Vasilios Sevastianos, Ioannis Vlachogiannakos, Alexandra Alexopoulou

**Affiliations:** 1 3rd Department of Internal Medicine & Liver Outpatient Clinic, Evangelismos General Hospital, 106 76 Athens, Greece; elgeladari@gmail.com (E.G.);; 2 Gastroenterology Department, Medical School, National & Kapodistrian University of Athens, Laiko General Hospital, 115 27 Athens, Greece; 3 Department of Nutrition & Dietetics, School of Health Sciences and Education, Harokopio University of Athens, 176 76 Kallithea, Greece; 4 Alexandra General Hospital, Gastroenterology, 115 28 Athens, Greece; 5 2nd Department of Internal Medicine & Research Laboratory, Medical School, National & Kapodistrian University of Athens, Hippokration General Hospital, 115 28 Athens, Greece

**Keywords:** myosteatosis, sarcopenia, liver cirrhosis, skeletal muscle wasting, survival

## Abstract

Background/Aims: Myosteatosis implies impaired muscle quality. The aim of the study was to investigate the association of myosteatosis with other muscle abnormalities and its role in the prognosis of liver cirrhosis (LC). Method: Skeletal muscle index (SMI) and myosteatosis were measured by computed tomography. Myosteatosis was defined as muscle radiodensity and evaluated according to dry body mass index (BMI). Median values and interquartile range were used for continuous and count (percentage) for categorical variables. Results: A total of 197 consecutive patients were included (age 61 (IQR 52–68); 67% male; MELD score 11 (interquartile range 7.5–16)). Myosteatosis was identified in 73.6% and sarcopenia in 44.6% of patients. Myosteatosis was positively associated with age (*p* = 0.024) and Child–Pugh (*p* = 0.017) and inversely associated with SMI (*p* = 0.026). Patients with myosteatosis exhibited lower 360-day survival (log-rank *p* = 0.001) compared to those without it. MELD (*p* < 0.001) and myosteatosis (*p* = 0.048) emerged as negative prognostic factors of survival in multivariate model. Individuals combining low muscle strength and impaired muscle quality and quantity displayed more advanced LC, impaired muscle performance, lower BMI (*p* < 0.001 each) and a three times higher mortality rate compared to those with low muscle quality alone. Conclusions: The presence of myosteatosis was associated with advanced age, low skeletal mass and more severe LC. Myosteatosis was associated with poor prognosis and may represent a prodromal phase of muscle degeneration before the development of sarcopenia.

## 1. Introduction

Myosteatosis is defined as increased fat infiltration or accumulation in skeletal muscle and implies compromised muscle quality. There are three potential phenotypes of adipose tissue distribution [1] in the muscles, i.e., inter-musculary, intra-musculary, and intra-myocellulary lipids. Myosteatosis is now considered a distinct entity from sarcopenia, but sarcopenia is also a component of its definition according to the updated European Working Group on Sarcopenia in Older People (EWGSOP-2) criteria [2]. Myosteatosis is associated with aging and mobility–disability consequences such as hip fractures, hospitalization, mortality and surgery in older adults [3,4]. It is also associated with high mortality rates in different clinical settings including critically ill patients with cancer, kidney or cardiovascular diseases [5,6,7,8]. In the cases of liver cirrhosis, myosteatosis is associated with minimal and overt hepatic encephalopathy and risk of falls [9,10,11] and has been incorporated into MELD as MELD–Sarco–Myo–HE score [12]. Moreover, myosteatosis has been implicated in the pre- and post-liver transplantation outcome [13,14,15]. Myosteatosis may be considered as a precursor of sarcopenia in the elderly [1] but its temporal correlation with muscle loss during the course of liver cirrhosis has not been documented.

Assessment of the cross-sectional surface area of axial and appendicular skeletal muscles by computed tomography (CT) at the level of the third lumbar vertebra (L3) is considered one of the most sensitive, widely used noninvasive approaches to evaluate muscle quantity and quality [16]. The evaluation of radiodensity in Hounsfield units (HU) is a measurement indicating the way X-rays pass through water. If density is lower than water, then there is evidence of fat. The lower the density, the higher the degree of myosteatosis. The correlation of HU evaluation with lipid content was confirmed by the direct evaluation of fat storage by muscle biopsy [17].

There is a debate in the literature whether myosteatosis is associated with severity of liver disease and whether there is an interaction between myosteatosis and low muscle mass or sarcopenia [18,19].

The aim of the present study was to investigate the interplay between muscle quality and quantity and the association of myosteatosis with the severity of liver cirrhosis, other muscle abnormalities such as low muscle mass and performance, and body adipose tissue composition. The role of myosteatosis in the prognosis of liver disease was also examined.

## 2. Materials and Methods

### 2.1. Study Population

This prospective study was conducted in a single tertiary center from May 2018 to December 2021. Patients with liver cirrhosis from the outpatient clinic and the wards of the Hippokration Hospital were included. The hospitalized patients were enrolled just before hospital discharge while they were in a stable condition without acute clinical events. Patients with alcoholic cirrhosis were included provided they had stopped consuming alcohol for at least one month before enrollment. The diagnosis of cirrhosis was based on liver histology and/or a combination of imaging, endoscopic and clinical findings. Cirrhosis was considered as decompensated in patients with history of ascites, variceal bleeding, hepatic encephalopathy and jaundice of non-obstructive cause (bilirubin >3 mg/dL for non-cholestatic and >10 mg/dL for cholestatic causes of cirrhosis). Patients with hepatocellular carcinoma or other extrahepatic malignancies, liver transplantation, immunosuppressive therapy other than corticosteroids human immunodeficiency virus infection and heart, kidney or respiratory failure were excluded from the study.

The study protocol was approved by the Hippokration Hospital ethical committee. All patients signed a written informed consent form before their inclusion in the study.

### 2.2. Clinical Data

Demographic and clinical data (such as age, gender, cause of liver cirrhosis) as well as laboratory parameters (including biochemical and clotting profile) were prospectively recorded. Based on these data, MELD score was calculated.

Body weight (BW) was measured using an electronic scale and standing height using a stadiometer (Seca 769 digital scale and Seca 220 stadiometer, respectively, Seca Medical Systems, Hamburg, Germany). Dry weight was calculated by subtracting 5% of the measured BW for mild ascites, 10% for moderate ascites and 15% for tense ascites, with an additional 5% subtracted if bilateral pedal edema was present, as suggested by European Association for the study of the liver [20]. Dry body mass index (BMI) (kg/m^2^) was also calculated.

### 2.3. Muscle Strength Assessment

Muscle strength for all included patients and controls was measured using a calibrated hydraulic hand dynamometer (Jamar Hydraulic Dynamometer, model 5030j1, Jamar Co., Duluth, MN, USA). Three handgrip measurements from each hand were recorded for each participant [21] and maximum measurements were used for statistical analysis. 

### 2.4. Muscle Mass Quantity and Quality Assessment

CT was performed within 7 days after enrollment. Muscle mass assessment was performed by measuring the muscle mass area at the level of L3, using appropriate software (SliceOmatic V4.3 software, Tomovision, Montreal, QC, Canada) as described by Georgiou A et al. [22]. Skeletal muscle was quantified using −29 to +150 Hounsfield Units (HU) range. The area was then adjusted to height in order to calculate skeletal mass index (SMI) (cm)^2^/height^2^ (in m^2^). Furthermore, an analysis of muscular, visceral and subcutaneous adipose tissue was also performed. Myosteatosis was defined as muscle radiodensity at L3 < 41 HU for patients with dry BMI < 24.9 kg/m^2^ and <33 HU for those with ≥25 kg/m^2^ [9,18]. The same software was utilized to calculate visceral adipose tissue index (VATI, cm^2^/m^2^) and subcutaneous adipose tissue index (SATI, cm^2^/m^2^).

### 2.5. Physical Performance Assessment

The short physical performance battery test (SPPB) was used to assess muscle performance. It includes the time the volunteer needs to perform five sits on a chair without using their hands, balance in three consecutive standing positions, and gait speed. Test scores range from 0 to 12 and SPPB score of ≤ 8 indicates decreased performance [23].

### 2.6. Sarcopenia Diagnosis

According to the updated EWGSOP-2 criteria [2], sarcopenia is diagnosed when low muscle strength and decreased muscle mass and/or quality (myosteatosis) are present. In the current study, low SMI cut-off values (<50 cm^2^/m^2^ for men and <39 cm^2^/m^2^ for women) set by Carey et al. [24] were used to identify patients with decreased muscle mass based on CT. If the patient had low muscle strength accompanied by decreased muscle mass and/or myosteatosis, they were classified as sarcopenic.

### 2.7. Follow-Up

The patients were prospectively followed-up during hospitalization and, if discharged, at the outpatient clinic, from electronic medical records and by telephone at 12 months.

### 2.8. Statistical Analysis

All data were analyzed using the statistical package SPSS (version 23.0; SPSS Inc., Chicago, IL, USA). Quantitative variables were expressed as median values and interquartile ranges (IQR) and categorical variables as count (percentage). Data were expressed as median and interquartile range (IQR) for continuous and count with percentage for categorical variables. Mann–Whitney *U* and Kruskal–Wallis tests were used for comparisons of continuous variables between groups and chi-squared test for categorical variables. A two-tailed *p*-value of less than 0.05 was considered statistically significant.

Factors associated with a *p* value of <0.05 in the univariate analysis as well as age and gender were entered in the multivariate model and non-significant factors were removed by a backward selection process.

Actuarial probabilities of death during follow-up were calculated by Kaplan–Meier method and compared between groups by log-rank test. Cox’s proportional hazards regression model was used to estimate risk factors that were associated with poor prognosis.

## 3. Results

A total of 197 consecutive patients (median age 61 (IQR 52–68), 67% male, MELD 11 (7.5–16), 60.9% with decompensated cirrhosis, 43.1%, 23.4% and 33.5% with alcoholic, viral and other causes of liver disease, respectively) were included in the study. Non-alcoholic steatohepatitis (NASH) or cryptogenic cirrhosis were diagnosed in 8.6% and 3% of patients, respectively. Dry BMI ≤25 kg/m^2^, >25 kg/m^2^ but ≤30 kg/m^2^ and >30 kg/m^2^ was measured in 41.1%, 39.6% and 19.3% of patients, respectively. Myosteatosis was identified in 73.6% and sarcopenia in 44.6% of the participants. Myosteatosis was present in all but three patients with sarcopenia (only three patients had low SMI and low handgrip without myosteatosis). Therefore, myosteatosis was identified in 96.5% of patients with sarcopenia.

Patients with myosteatosis vs. those without were older (*p* = 0.004) and had more often alcoholic etiology (*p* = 0.028), decompensated cirrhosis (*p* < 0.001), and history of hepatic encephalopathy (*p* = 0.013) (Table 1). Moreover, patients with myosteatosis compared to those without displayed lower dry BMI (*p* = 0.001), lower skeletal muscle mass (*p* < 0.001) and decreased muscle performance (*p* < 0.001). Gender, VATI and SATI did not produce different effects between the two groups (Table 1). Myosteatosis was present in 89.5% of patients with low SMI.

In multivariate analysis, after adjusting for age, gender, dry BMI, Child–Pugh score, SMI, handgrip and SPPB, only advanced age (*p* = 0.024), low SMI (*p* = 0.026) and advanced Child–Pugh (*p* = 0.017) appeared to be associated with myosteatosis (multivariate 1). If cirrhosis status (dichotomized as decompensated or non-decompensated) replaced Child–Pugh score, then low SMI (*p* = 0.013) and decompensated cirrhosis (*p* = 0.009) were associated with the presence of myosteatosis (multivariate 2). MELD score was not associated with the presence of myosteatosis in multivariate analysis (multivariate 3) (Table 2).

### 3.1. Survival

**Figure 1:**
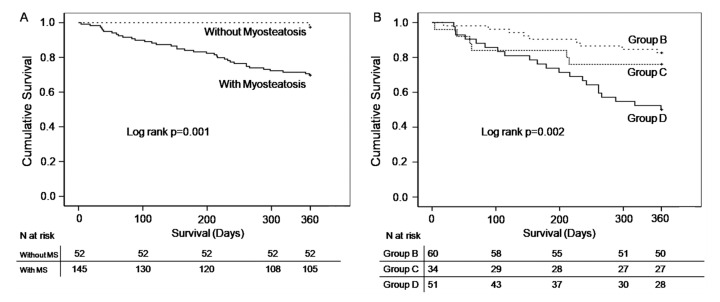
Kaplan–Meier survival curves for patients with myosteatosis vs. those without (**A**); Kaplan–Meier survival curves for the 3 phenotypes according to the extent of muscle abnormalities (myosteatosis alone (group B); myosteatosis combined by low handgrip strength (group C *); myosteatosis combined by low handgrip strength and low skeletal mass index (group D *) (**B**). * Groups C and D had sarcopenia according to the updated EWGSOP-2 criteria [2].

After assessing Kaplan–Meier survival curves at 360 days, patients with myosteatosis displayed increased mortality rate compared to those without (no patient died in those without myosteatosis) (log-rank *p* = 0.001) (Figure 1A). Cox’s univariate analysis showed that the variables exhibiting association with mortality were age (*p* = 0.002), presence of myosteatosis (*p* = 0.008), MELD score (*p* < 0.001), low SMI (*p* < 0.001) and low SATI (*p* = 0.030). In multivariate analysis (after adjustment for age, gender, dry BMI, MELD, low SMI and SATI), only MELD score (HR 4.911, 95% CI 2.390–10.094, *p* < 0.001) and the presence of myosteatosis (HR 7.778, 95% CI 1.022–59.206, *p* = 0.048) emerged as independent, prognostic factors of mortality (Table 3). All deaths were due to liver-related causes.

### 3.2. Study of Groups Composed According to the Extent of Muscle Abnormalities

Taking into consideration three parameters, i.e., presence of myosteatosis, low handgrip strength and low SMI, 194 patients were classified into four groups: neither myosteatosis nor sarcopenia in 25.3% (group A), myosteatosis alone (with normal handgrip and normal SMI) in 30.9% (group B), sarcopenia according to low handgrip strength and myosteatosis (but normal SMI) in 17.5% (group C) and sarcopenia according to low handgrip, myosteatosis and low SMI in 26.3% (group D). Only three cases exhibited low handgrip and low SMI (sarcopenia) without myosteatosis, so no group was composed from these three patients.

There was a sequential ascending order across the groups A, B, C and D in age (56 (50–63.5), 57.5 (51.2–66), 62.5 (57–69.5) and 67 (59–72.5) years, respectively; *p* < 0.001) and severity of LC as it was documented by the increasing rate of decompensated cirrhosis (32.7%, 58.3%, 73.5% and 80.4%, respectively; *p* < 0.001) and Child–Pugh (5 (5–7), 7 (5–9), 7 (6–8) and 9 (7–10), respectively; *p* < 0.001). Similar but not so well-arranged results were demonstrated in MELD score (Table 4). In contrast, there was a sequential descending order across the groups in muscle performance (SPPB) (*p* < 0.001) values. Similar but not so well-arranged results were illustrated in dry BMI, handgrip force, SATI and SMI (*p* < 0.001 for all the above). No difference among groups was demonstrated in gender and VATI.

Group D had significantly more advanced liver disease (according to MELD and Child–Pugh score), lower BMI, muscle performance and SATI compared to group C. Patients in group D were older, more often of alcoholic etiology, had significantly more advanced liver disease, lower BMI, functionality and SATI compared to groups B and A. Patients in Group C had similar parameters to group B but they had lower BMI and muscle performance compared to those of group B. Patients in group C were older, had significantly more advanced liver disease lower muscle functionality and SATI compared to those of group A. Group B had significantly more advanced liver disease, lower BMI and SATI compared to those of group A (Table S1).

Considering the Kaplan–Meier curve at 360 days, in groups exhibiting myosteatosis alone (group B) or with myosteatosis plus more extended muscle abnormalities (C and D) (no patient died in group A), patients of group D had a higher mortality rate compared to those of group B (log-rank *p* = 0.001) but not C (log-rank *p* = 0.068) (log-rank *p* = 0.002 in overall) (Figure 1B). According to the Cox’s regression analysis, after adjustment for age and gender, patients in group C had similar risk of death to those of group B (reference group) (*p* = 0.530). However, mortality was three times higher in those of group D compared to those of group B (HR 3.097 (1.338–7.169), *p* = 0.008) (Table 5).

## 4. Discussion

The presence of myosteatosis was associated with aging, more severe liver cirrhosis and history of hepatic encephalopathy, lower skeletal muscle mass, performance and BMI in this cohort of patients with cirrhosis of various etiologies, more than half decompensated. Patients with myosteatosis displayed poor outcome even after adjusting for significant covariates. When patients with myosteatosis were divided according to the severity level of muscle aberrations, individuals combining low muscle strength, impaired quality and quantity were older and displayed more often alcoholic etiology, advanced liver disease, lower BMI, more impaired muscle quality and performance and higher mortality rate compared to those with low muscle quality alone.

Myosteatosis was detected in 74% of our patients with cirrhosis even in the absence of sarcopenia. Concomitant presence of myosteatosis occurred in 96.5% patients with sarcopenia (defined by the most recent EWGSOP-2 criteria) [2] and in 89.5% of those with low muscle mass (SMI), displaying an interconnection between impaired muscle quality and quantity. It was also illustrated that myosteatosis was associated with the presence of hepatic encephalopathy, a finding that was previously reported in the literature [9,10,11]. It was suggested that hyperammonemia resulting in skeletal muscle ammonia uptake promoted skeletal muscle mitochondrial dysfunction, decreased lipid oxidation and finally led to lipid deposition in muscles [25]. In addition, systemic inflammation and oxidative stress usually accompany liver cirrhosis and are associated with metabolic dysfunction of skeletal muscle, impaired muscle protein synthesis, turnover and function [26,27]. Most patients with liver cirrhosis live a sedentary life with restricted daily physical activity. In addition, they may experience decreased appetite due to salt restriction and alcohol consumption, early satiety due to ascites, and impaired gastric and intestinal motility [28]. Measuring dietary intake is difficult in clinical practice and accurate assessments remain unclear in patients experiencing complications of cirrhosis. All the above along with aging may result in malnutrition, increased muscle catabolism, muscle atrophy and replacement of muscle by adipose tissue.

In the present study, myosteatosis was independently associated with higher Child–Pugh score or decompensated cirrhosis status but not with MELD score. Child–Pugh score includes two clinical components, i.e., ascites and hepatic encephalopathy, which are not included in MELD score. It is therefore reasonable that patients with clinical characteristics of decompensated cirrhosis (ascites, hepatic encephalopathy) have reduced physical activity and diminished food intake, which along with hyperammonemia decrease body weight and deteriorate muscle robustness.

There is a correlation between myosteatosis and obesity or total body fat percentage in patients with non-alcoholic fatty liver disease without cirrhosis [29,30]. In addition, in patients with morbid obesity and NASH, muscle fat content was reported high, but no low muscle mass was observed [31]. The population investigated in the above studies was totally different from that in current study. It concerned individuals with NASH usually without cirrhosis [29,30,31]. In the present study, only patients with cirrhosis were included and the vast majority of them had alcoholic or viral etiology. Only a small part had NASH or cryptogenic cirrhosis. Moreover, we did not demonstrate any correlation of myosteatosis with increased visceral fat deposition or elevated body weight. On the contrary, we detected a link between the presence of myosteatosis and reduced dry body weight implying that mechanisms other than insulin resistance or fat accumulation might explain the high rate of myosteatosis in end-stage liver disease. There is evidence of increasing global burden of NASH-relating cirrhosis with established “pre-existing” obesity-related myosteatosis and alterations in muscle and adipose tissue. Hence, it remains to be seen whether the above-mentioned relationships between myosteatosis and liver-related outcomes are upheld in these patient populations.

We used CT imaging to assess myosteatosis and we selected the muscle attenuation cut-offs proposed by previous investigators [9,32]. CT is a widely available method; it can be acquired as part of clinical routine in patient care and was reported as the best option for estimating myosteatosis [18,27,32,33]. However, CT cannot directly measure the location of fat deposition or lipid droplets in the muscle and cannot differentiate among potential phenotypes of fat distribution [1,34]. Therefore, the type and location of muscle fat infiltration occurring in different settings of liver disease need to be further examined.

We also demonstrated that muscle changes were increasing in accordance with liver disease severity. Four main phenotypes according to the extent of muscle changes were exhibited: neither MS nor sarcopenia, myosteatosis alone, myosteatosis combined by decreased muscle force (sarcopenia according to EWGSOP-2) [2] and myosteatosis coexisting with decreased muscle strength and muscle wasting (sarcopenia according to EWGSOP-2) [2]. The magnitude of muscle abnormalities aligned with the severity of liver cirrhosis, increasing age, and waning in muscle force and functionality. Muscle attenuation level (degree of myosteatosis) was better in the myosteatosis alone phenotype but worsened further in phenotype where myosteatosis coexisted with decreased muscle strength and mass loss. Only three persons had low handgrip strength and muscle mass (sarcopenia) without myosteatosis, showing that myosteatosis usually precedes subsequent muscle abnormalities in this clinical setting. The presence of myosteatosis alone in earlier stages as opposed to additive muscle defects in late stages of liver cirrhosis may imply that myosteatosis antedated muscle force decline and sarcopenia during the natural course of liver cirrhosis. On the other hand, myosteatosis acted synergistically with muscle mass loss and impaired performance in late stages of liver cirrhosis.

Previous investigators have reported that the presence of myosteatosis is a poor prognostic factor for liver cirrhosis outcome [12,13,35]. In the present study, no patient died during one year-follow-up in non-myosteatosis group. In addition, we demonstrated that myosteatosis is associated with high risk of death after adjusting for multiple covariates. When we divided patients into groups according to the extent of muscle changes, we discovered that mortality risk was increasing as the muscle quantity, quality and performance worsened. More specifically, patients with all three changes (myosteatosis, low muscle force and muscle mass loss) had more than three times higher risk of death than those with myosteatosis alone.

Our study acknowledges particular strengths. The sample is large and well studied, dry body weight has been used, the methods of measuring muscle mass, quality and body composition of adipose tissue are sophisticated, and those of diagnosing sarcopenia are based on the most recently updated criteria.

The limitations of the study include the lack of muscle biopsies for the location of fat deposition and the absence of longitudinal data to better investigate the evolution of myosteatosis during the course of liver cirrhosis from compensated to decompensated.

In conclusion, the presence of myosteatosis was positively associated with age and severity of liver cirrhosis and it was inversely associated with skeletal muscle mass, performance, and BMI. The magnitude of muscle abnormalities in liver cirrhosis is proportional to age and severity of liver disease. Myosteatosis alone is present in earlier stage of the disease and younger age and may indicate a prodromal phase of muscle degeneration before the development of sarcopenia. Myosteatosis is a poor prognostic factor of outcome after adjusting for multiple covariates. The combination of multiple muscle abnormalities has an unfavorable effect on survival.

## Figures and Tables

**Table 1:** Comparison of demographics, clinical characteristics between patients with and without myosteatosis (N = 197). Median values and interquartile range are used for continuous and count (percentage) for categorical variables.

Parameter	Total Patients Ν = 197	Without Myosteatosis Ν = 52	With Myosteatosis Ν = 145	*p* Value
Age (years)	61.0 (52.0–68.0)	56.0 (50.0–64.0)	63.0 (55.0–70.0)	0.004
Gender (% male)	132 (67)	40 (76.9)	92 (63.4)	0.076
Etiology (%)				0.028
Alcohol	85 (43.1)	20 (38.5)	65 (44.8)	
Viral	46 (23.4)	19 (36.5)	27 (18.6)	
Other	66 (33.5)	13 (25.0)	53 (38.6)	
Dry BMI (kg/m^2^)	25.90 (22.28–28.71)	26.60 (25.60–29.81)	24.70 (21.79–28.60)	0.001
Decompensated cirrhosis (%)	120 (60.9)	19 (36.5)	101 (69.7)	<0.001
MELD score	11.00 (7.50–16.00)	9.00 (7.00–12.00)	12.00 (8.00–16,25)	0.009
Child–Pugh score	7.0 (5.0–9.0)	5.0 (5.0–7.0)	8.0 (6.0–9.0)	<0.001
History of hepatic encephalopathy (%)	41 (20.8)	4 (7.7)	37 (25.5)	0.013
Handgrip strength (kg)	27.0 (19.0–34.0)	30.5 (25.0–39.5)	26.0 (18.0–32.0)	0.001
Skeletal mass index (cm^2^/m^2^)	47.35 (41.70–54.13)	54.11 (48.95–57.42)	45.12 (40.25–51.27)	<0.001
Low skeletal mass index (N%)	86 (43.7)	9 (17.3)	77 (53.1)	<0.001
Short Physical Performance Battery	10.0 (8.0–12.0)	11.5 (10.0–12.0)	10.0 (6.0–11.0)	<0.001
Visceral Adipose Tissue Index (cm^2^/m^2^)	51.04 (28.48–66.46)	47.63 (27.76–62.69)	51.68 (27.30–67.36)	0.242
Subcutaneous Adipose Tissue Index (cm^2^/m^2^)	58.12 (35.96–87.37)	64.70 (48.31–89.51)	56.22 (34.15–86.43)	0.114

**Table 2:** Factors associated with presence of myosteatosis in univariate and multivariate analysis (3 models) in all 197 patients.

Parameter	Univariate Analysis	*p* Value	Model 1	P1 Value	Model 2	P2 Value	Model 3	P3 Value
Age (years)	1.047 (1.016–1.080)	0.003	1.047 (1.006–1.090)	0.024				
Gender (% male)	1.920 (0.927–3.978)	0.079						
Handgrip strength	0.952 (0.922–0.983)	0.003						
Decompensated vs. compensated cirrhosis	3.987 (2.048–7.762)	<0.001			3.129 (1.336–7.328)	0.009		
MELD score	1.105 (1.031–1.184)	0.005						
Child–Pugh score	1.400 (1.167–1.680)	<0.001	1.320 (1.052–1.657)	0.017				
Skeletal mass index (cm^2^/m^2^)	0.920 (0.883–0.957)	<0.001	0.937 (0.885–0.992)	0.026	0.932 (0.882–0.985)	0.013	0.943 (0.893–0.996)	0.036
Short Physical Performance Battery	0.695 (0.577–0.839)	<0.001					0.777 (0.619–0.976)	0.030

From the parameters evaluating the severity of liver cirrhosis, only one was entered in each multivariate model (Model 1, Child–Pugh score; Model 2, Decompensated vs. compensated cirrhosis; Model 3, MELD score); P1, P2 and P3 values correspond to Model 1, 2 and 3, respectively. Only statistically significant results are mentioned in multivariate analysis.

**Table 3:** Factors predicting 360-day mortality in univariate and multivariate analysis in 197 patients.

Parameter	Univariate Analysis	*p* Value	Multivariate Analysis	*p* Value
	HR (95% CI)		HR (95% CI)	
Age * (years)	1.052 (1.018–1.085)	0.002		
Gender * (% male)	1.243 (0.633–2.442)	0.527		
Dry Body Mass Index *	0.902 (0.839–0.970)	0.005		
MELD score *	1.138 (1.096–1.182)	<0.001	4.911 (2.390–10.094)	<0.001
Skeletal mass index * (cm^2^/m^2^)	0.915 (0.880–0.951)	<0.001		
Short Physical Performance Battery (SPPB)	0.763 (0.704–0.826)	<0.001		
Visceral Adipose Tissue Index (VATI) cm^2^/m^2^	0.997 (0.985–1.009)	0.601		
Subcutaneous Adipose Tissue Index * (SATI) cm^2^/m^2^	0.989 (0.979–0.999)	0.030		
Myosteatosis *	14.042 (1.925–102.400)	0.008	7.778 (1.022–59.206)	0.048

* Variables entering in multivariate analysis.

**Table 4:** Comparison of demographics and clinical characteristics among 4 groups according to the extent of muscle abnormalities (194 patients).

	Group A Neither Myosteatosis Nor Sarcopenia (N = 49)	Group B Myosteatosis Alone (N = 60)	Group C Myosteatosis Combined by Low Handgrip * (N = 34)	Group D Myosteatosis Combined by Low Handgrip and Low SMI * (N = 51)	*p*
Age	56.0 (50.0–63.5)	57.5 (51.25–66.00)	62.5 (57.0–69.5)	67.00 (59.00–72.50)	<0.001
Gender (Male, N, %)	37 (75.5)	40 (66.7)	17 (50.00)	35 (68.62)	0.109
Dry BMI	26.67 (25.61–29.98)	24.4 (22.04–28.83)	28.15 (24.08–34.05)	23.28 (20.80–26.41)	<0.001
Etiology (N, %):					0.038
Alcoholic	20 (40.81)	24 (40)	17 (50.00)	24 (47.05)	
Viral	17 (34.69)	17 (28.33)	6 (17.64)	4 (7.80)	
Other	12 (24.48)	19 (31.66)	11 (32.45)	23 (45.09)	
Decompensated cirrhosis (N, %)	16 (32.65)	35 (58.33)	25 (73.52)	41 (80.39)	<0.001
ΜELD score	9.0 (7.0–10.75)	11.0 (7.00–14.0)	10.0 (7.00–15.75)	15.0 (11.50–21.50)	<0.001
Child–Pugh score	5.0 (5.0–7.0)	7.0 (5.0–9.0)	7.0 (6.0–8.0)	9.0 (7.0–10.0)	<0.001
Handgrip strength	32.0 (24.50–40.0)	34.00 (30.0–38.00)	18.50 (15.50–27.25)	20.0 (14.0–25.0)	<0.001
SMI	54.20 (49.72–57.48)	46.84 (42.25–53.03)	51.03 (44.38–57.82)	40.50 (31.57–45.54)	<0.001
SPPB	12.0 (10.0–12.0)	11.0 (10.0–12.0)	9.0 (6.75–11.0)	8.00 (3.75–10.00)	<0.001
VATI	45.38 (27.97–62.28)	50.60 (28.85–80.08)	54.78 (35.72–96.31)	47.43 (26.16–63.81)	0.194
SATI	66.25 (48.62–92.32)	58.10 (36.35–87.65)	87.27 (48.62–124.09)	44.36 (25.90–66.70)	<0.001
Mean attenuation in HU	37.34 (33.94–40.35)	30.66 (26.75–35.65)	27.98 (23.30–31.15)	27.83 (22.50–32.35)	<0.001

* Groups C and D had sarcopenia according to the updated EWGSOP-2 criteria [2]; HU, Hounsfield Units; SATI, subcutaneous adipose tissue index; SMI, skeletal mass index; SPPB, short physical performance battery test; VATI, visceral adipose tissue index; Kruskal–Wallis test (*p*) and x^2^ test were used for continuous and categorical variables, respectively.

**Table 5:** Hazard ratios for death according to extent of muscle abnormalities. Crude and adjusted values by the use of Cox’s regression analysis.

	Univariate Analysis		Multivariate Analysis	
	HR (95% CI)	*p*	HR (95% CI)	*p*
Age (per 1 year)	1.040 (1.006–1.075)	0.022	1.020 (0.984–1.057)	0.275
Gender (men vs. women)	0.917 (0.465–1.811)	0.804	0.800 (0.394–1.622)	0.536
Myosteatosis alone (group B)	Reference group		Reference group	
Myosteatosis combined by low handgrip (group C *)	1.537 (0.547–4.318)	0.415	1.397 (0.492–3.693)	0.530
Myosteatosis combined by low handgrip and low skeletal muscle index (group D *)	3.505 (1.603–7.663)	0.002	3.097 (1.338–7.169)	0.008

No patient died in no muscle abnormality group (group A); * Groups C and D had sarcopenia according to the updated EWGSOP-2 criteria [2].

## Data Availability

Data are available on request.

## Supplementary Materials

The following supporting information can be downloaded at: https://www.mdpi.com/article/10.3390/jcm12093332/s1, Table S1: Comparison of demographics and clinical characteristics among 4 groups according to the extent of muscle abnormalities (patients 194).
