# Supplementary material for: The Presence of Myosteatosis Is Associated with Age, Severity of Liver Disease and Poor Outcome and May Represent a Prodromal Phase of Sarcopenia in Patients with Liver Cirrhosis
Source: J Clin Med. 2023 May 7;12(9):3332. doi: 10.3390/jcm12093332 (PMC10179726; doi:10.3390/jcm12093332)
Supplement: Supplementary file 1 [file jcm-12-03332-s001.zip › jcm-2369682-supplementary.pdf]

Table S1. Comparison of demographics and clinical characteristics among 4 groups according to the extent of muscle abnormalities (patients 194)

	**Group A Neither MS nor sarcopenia** (N=49)	**Group B MS alone** (N=60)	**Group C MS combined by low handgrip*** (N=34)	**Group D MS combined by** low **handgrip & low SMI*** (N=51)	P_1	P_2	P_3	P_4	P_5	P_6	P
**Age**	56.0 (50.0-63.5)	57.5 (51.25-66.00)	62.5 (57.0-69.5)	67.00 (59.00-72.50)	0.176	0.009	<0.001	0.111	0.003	0.121	< 0.001
**Gender (Male, N, %)**	37 (75.5)	40 (66.7)	17 (50.0)	35 (68.6)	0.313	0.017	0.443	0.112	0.826	0.084	0.109
**Dry BMI**	26.67 (25.61-29.98)	24.4 (22.04-28.83)	28.15 (24.08-34.05)	23.28 (20.80-26.41)	0.004	0.520	<0.001	0.018	0.020	<0.001	<0.001
**Aetiology (N, %):**<br>**- Alcoholic**<br>**- Viral**<br>**- Other**	 20 (40.8)<br>17 (34.7)<br>12 (24.5)	 24 (40)<br>17 (28.3)<br>19 (31.7)	 17 (50.0)<br>6 (17.6)<br>11 (32.4)	 24 (47.1)<br>4 (7.8)<br>23 (45.1)	0.656	0.231	0.003	0.469	0.021	0.282	0.038
**Decompensated cirrhosis (N, %)**	16 (32.7)	35 (58.3)	25 (73.5)	41 (80.4)	0.008	< 0.001	< 0.001	0.141	0.013	0.457	< 0.001
**MELD score**	9.0 (7.0-10.75)	11.0 (7.00-14.0)	10.0 (7.00-15.75)	15.0 (11.50-21.50)	0.241	0.184	< 0.001	0.743	< 0.001	0.017	< 0.001
**Child-Pugh score**	5.0 (5.0-7.0)	7.0 (5.0-9.0)	7.0 (6.0-8.0)	9.0 (7.0-10.0)	0.01	0.002	< 0.001	0.453	0.002	0.043	< 0.001
**Handgrip strength**	32. 0 (24.50-40.0)	34.00 (30.0-38.00)	18.50 (15.50-27.25)	20.0 (14.0-25.0)	0.240	< 0.001	< 0.001	< 0.001	< 0.001	0.689	< 0.001
**SMI**	54.20 (49.72-57.48)	46.84 (42.25-53.03)	51.03 (44.38-57.82)	40.50 (31.57-45.54)	< 0.001	0.189	< 0.001	0.057	< 0.001	< 0.001	< 0.001
**SPPB**	12.0 (10.0-12.0)	11.0 (10.0-12.0)	9.0 (6.75-11.0)	8.00 (3.75-10.00)	0.416	< 0.001	< 0.001	< 0.001	< 0.001	0.036	< 0.001
**VATI**	45.38 (27.97-62.28)	50.60 (28.85-80.08)	54.78 (35.72-96.31)	47.43 (26.16-63.81)	0.171	0.079	0.893	0.504	0.210	0.101	0.194
**SATI**	66.25 (48.62-92.32)	58.10 (36.35-87.65)	87.27 (48.62-124.09)	44.36 (25.90-66.70)	0.155	0.151	< 0.001	0.008	0.008	< 0.001	< 0.001
**Mean HU attenuation**	37.34 (33.94-40.35)	30.66 (26.75-35.65)	27.98 (23.30-31.15)	27.83 (22.50-32.35)	< 0.001	< 0.001	< 0.001	0.026	0.026	0.950	< 0.001

*Groups C and D had sarcopenia according to the updated EWGSOP-2 criteria ([2]); HU, Hounsfield Units; MS myosteatosis; SATI, subcutaneous adipose tissue index; SMI, skeletal mass index; SPPB, short physical performance battery test; VATI, visceral adipose tissue index

P_1: corresponds to the comparison between groups A B; P_2 corresponds to the comparison between groups A and C; P_3 corresponds to the comparison between groups A and D; P_4 corresponds to the comparison between groups B and C; P_5 corresponds to the comparison between groups B and D; P_6 corresponds to the comparison between groups C and D

Mann-Whitney (P_1, P_2, P_3, P_4, P_5, P_6) and Kruskal Wallis tests (P) were used for continuous variables and for x^2 test was used for categorical valuables
